# Raloxifene and Desmethylarzoxifene Block Estrogen-Induced Malignant Transformation of Human Breast Epithelial Cells

**DOI:** 10.1371/journal.pone.0027876

**Published:** 2011-11-29

**Authors:** Irida Kastrati, Praneeth D. Edirisinghe, L-P-Madhubani P. Hemachandra, Esala R. Chandrasena, Jaewoo Choi, Yue-Ting Wang, Judy L. Bolton, Gregory R. J. Thatcher

**Affiliations:** Department of Medicinal Chemistry and Pharmacognosy, College of Pharmacy, University of Illinois at Chicago, Illinois, United States; Instituto de Investigación Sanitaria INCLIVA, Spain

## Abstract

There is association between exposure to estrogens and the development and progression of hormone-dependent gynecological cancers. Chemical carcinogenesis by catechol estrogens derived from oxidative metabolism is thought to contribute to breast cancer, yet exact mechanisms remain elusive. Malignant transformation was studied in MCF-10A human mammary epithelial cells, since estrogens are not proliferative in this cell line. The human and equine estrogen components of estrogen replacement therapy (ERT) and their catechol metabolites were studied, along with the influence of co-administration of selective estrogen receptor modulators (SERMs), raloxifene and desmethyl-arzoxifene (DMA), and histone deacetylase inhibitors. Transformation was induced by human estrogens, and selectively by the 4-OH catechol metabolite, and to a lesser extent by an equine estrogen metabolite. The observed estrogen-induced upregulation of CYP450 1B1 in estrogen receptor negative MCF-10A cells, was compatible with a causal role for 4-OH catechol estrogens, as was attenuated transformation by CYP450 inhibitors. Estrogen-induced malignant transformation was blocked by SERMs correlating with a reduction in formation of nucleobase catechol estrogen (NCE) adducts and formation of 8-oxo-dG. NCE adducts can be formed consequent to DNA abasic site formation, but NCE adducts were also observed on incubation of estrogen quinones with free nucleotides. These results suggest that NCE adducts may be a biomarker for cellular electrophilic stress, which together with 8-oxo-dG as a biomarker of oxidative stress correlate with malignant transformation induced by estrogen oxidative metabolites. The observed attenuation of transformation by SERMs correlated with these biomarkers and may also be of clinical significance in breast cancer chemoprevention.

## Introduction

With an average lifetime risk of 8–10%, breast cancer is the most common malignancy in women in the Western world. Longer exposure to estrogens predisposes women to develop hormone-dependent gynecological malignancies. A central role for circulating hormones in breast cancer development is further supported by the marked reduction in cancer incidence after surgical or chemical ovariectomy. Direct action of estrogen has been shown to cause malignant transformation of normal breast epithelial cells in culture, even when these cells are unresponsive to classical estrogen receptor (ER) mediated proliferation [1].

Malignant phenotypes of the breast arise as a result of a series of mutations, most likely in genes associated with tumor suppressor, oncogene, DNA repair, or endocrine function. Chemical carcinogenesis resulting from estrogen oxidative metabolism to quinoid metabolites is associated with electrophilic and oxidative damage to DNA. Typical DNA damage includes formation of stable adducts, nucleobase oxidation, formation of abasic sites, single strands breaks, mutations such as G→T transversions, loss of heterozygosity, and epigenetic changes.

**Figure 1:**
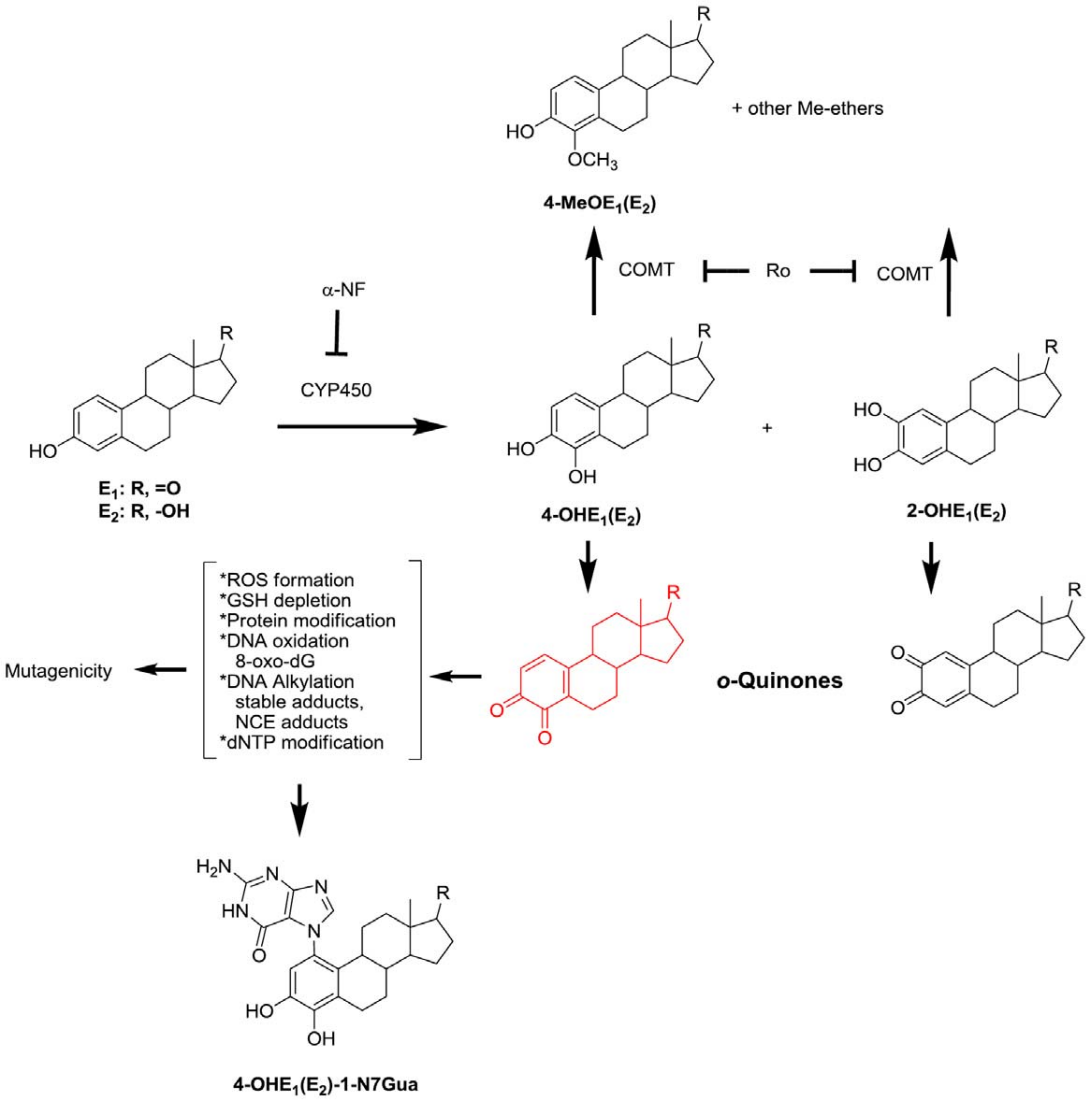
Estrogens (E_1_ and E_2_) undergo oxidative metabolism to C2 and C4 catechols. Catechols are further oxidized to form *o*-quinones. Based on *t_1/2_* and reactivity, the most mutagenic species is 4-OHE_1/2_ and its further oxidized species. 4-OHE-3,4-*o*-quinone (shown in red) can redox cycle to form ROS, oxidize DNA (measured as 8-oxo-dG), alkylate DNA (form abasic sites, and stable adducts), and chemically modify other important macromolecules such as dNTP, GSH, and cellular proteins.

Formation of the catechol estrogens, 2-OHE and 4-OHE, is catalyzed by the action of CYP450 enzymes, most importantly CYP450 1B1-mediated formation of the genotoxic 4-OHE (Figure 1). DNA modification by the carcinogenic 4-OHE quinone is known to cause depurination leading to abasic sites and nucleobase catechol estrogen (NCE) adducts. In contrast, DNA adducts formed by 2-OHE quinone are proposed to be chemically stable, not generating appreciable amounts of abasic sites [2]. Together with receptor-mediated, or hormonal carcinogenesis, by estrogen and estrogen metabolites, the genotoxic *o*-quinone metabolites of estradiol (E_2_) and estrone (E_1_) are argued to be initiators and promoters of breast cancer via chemical carcinogenesis (Figure 1).

Estrogen replacement therapy (ERT) remains a cornerstone of contemporary women's healthcare, despite outcomes from the Women's Health Initiative clinical trials that reported the elevated risks of breast and lung cancer associated with ERT [3], [4], [5]. These trials also confirmed the benefits of ERT including reductions in osteoporotic fractures, and adverse postmenopausal symptoms, highlighting the need for new ERT agents with minimized cancer risk. Selective estrogen receptor modulators (SERMs), such as the benzothiophene SERMs, raloxifene and arzoxifene, have the potential to fill such a need [6]. An ideal SERM would provide estrogenic agonist activity in bone, the cardiovascular system, and the central nervous system, whilst exerting antagonist activity in the breast and uterus [7], [8], [9], [10]. It was therefore of interest to study malignant transformation of human mammary epithelial cells induced by the components of ERT and modulated by benzothiophene SERMs. Since the actions of histone deacetylase (HDAC) and SERMs has been linked and HDACs are in clinical trials for breast cancer [11], [12], HDAC inhibitors (HDACIs) were also of interest.

The non-tumorigenic MCF-10 cell line has the morphological characteristics of normal breast epithelial cells [13], [14], [15]; in response to extended treatment with E_2_, anchorage-independent growth was induced, a phenotype of malignant transformation, strongly correlated with tumorigenicity [16], [17]. CYP450 1B1 was implicated since protein expression was upregulated by estrogen treatment. The objectives of this work were: firstly to study malignant transformation of a mammary cell line, modeling estrogen-induced chemical carcinogenesis resulting from ERT and to examine the influence of SERM and HDACI co-administration; and, secondly to examine the correlation of malignant transformation with biomarkers of cellular electrophilic and oxidative stress. The biomarkers investigated, NCE adducts and 8-oxo-dG, were elevated by estrogens and attenuated by SERM co-treatment.

## Results

### Malignant transformation of MCF-10A cells induced by estrogens and metabolites

**Figure 2:**
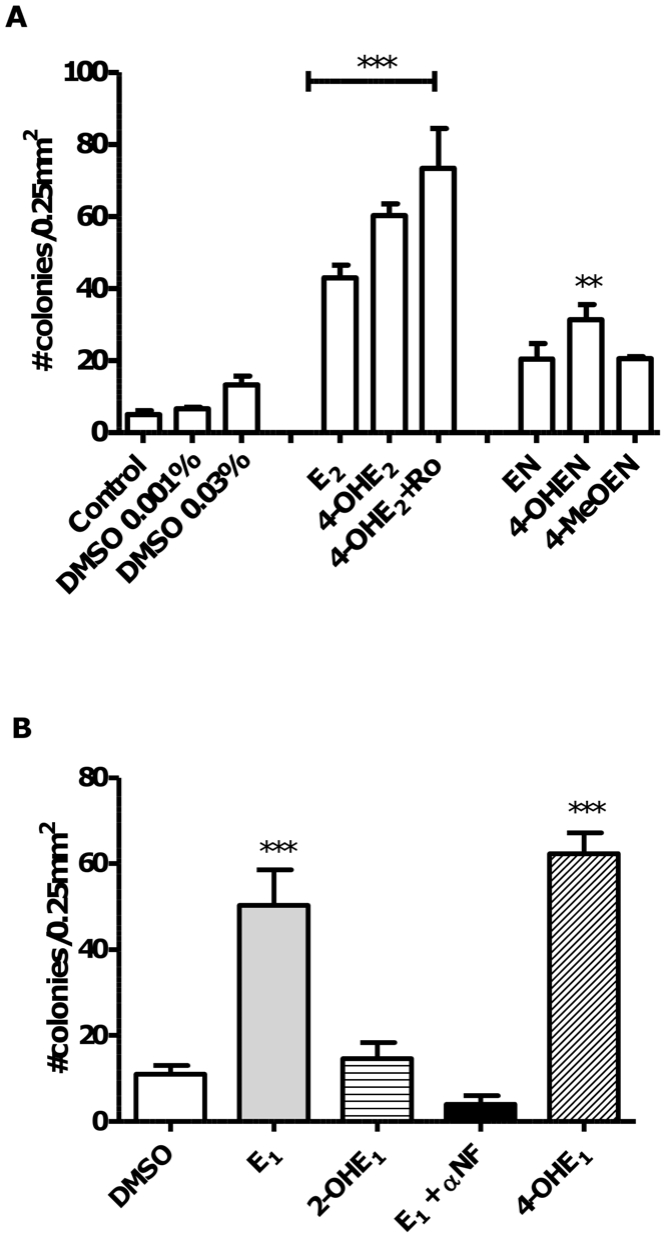
Malignant transformation induced by endogenous and equine estrogens: MCF-10A cellular transformation induced by estrogens and metabolites was measured as anchorage-independent growth in soft agar after four weeks. **A**. Cells were treated with estrogens or metabolites (1 µM) and COMT inhibitor Ro 41-0960 (Ro, 3 µmol) for 4 weeks before transfer to soft agar. Using one-way ANOVA with Dunnett's post test: *** p<0.001; ** p<0.01 versus DMSO control. **B**. MCF-10A cellular transformation induced by E_1_ with or without the CYP450 1B1 inhibitor αNF (3 µM), and a comparison between transformation potency of 2-OHE_1_ versus 4-OHE_1_ in MCF-10A cells, 1 µM each. Using one-way ANOVA with Dunnett's post test: *** p<0.001; ** p<0.01 versus DMSO control.

Treatment of MCF-10A immortalized, human breast epithelial cell cultures with E_2_ or the catechol metabolite 4-OHE_2_, for four weeks, led to significantly increased cellular transformation as assessed by anchorage-independent colony growth performed in soft-agar for a further four week-period (Figure 2A). The COMT (catechol *O*-methyl transferase) inhibitor, Ro 41-0960, would be expected to block methylation and deactivation of the catechol estrogen (Figure 1) and to increase the extent of cellular transformation, however, the observed increase, when the inhibitor was co-administered with 4-OHE_2_ did not reach significance compared to 4-OHE_2_ alone. In a similar way, cells were treated with equilenin (EN), and its oxidative metabolite formed on CYP450 1B1 action, 4-OHEN (4-hydroxyequilenin). Co-treatment of 4-OHEN with COMT inhibitor, proved to be cytotoxic to MCF-10A cells over the 4-week treatment period, therefore, in the case of the equine estrogens, the product of COMT mediated methylation, 4-MeOEN (4-methoxyequilenin), was studied. 4-OHEN caused significant cellular transformation compared to vehicle control, whereas EN and 4-MeOEN did not reach significance. The equine estrogens were less potent inducers of cellular transformation than human estrogens as measured by anchorage-independent colony growth.

**Figure 3:**
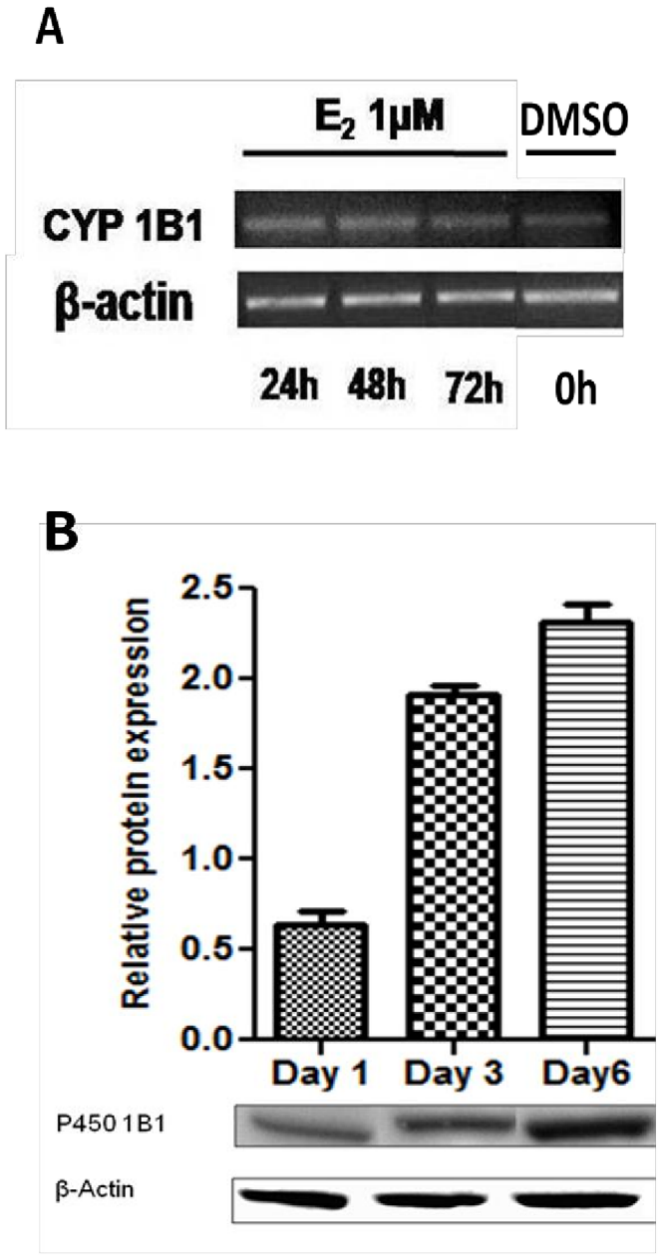
Induction of CYP450 1B1 expression in MCF-10A cells by E_2_ (1 µM). Cells were collected at different time points (24 h, 2, 3 and 6 days). Increased CYP450 1B1 mRNA levels **A**. from E_2_ treatment measured with real-time PCR, correlate with protein levels **B**. extracted and analyzed by western blot using anti CYP450 1B1 rabbit polyclonal primary antibody. Representative blots are shown of the 55 kDa immunoreactive band. Intensity of the bands was normalized to β-actin (N = 6) showing mean and s.e.m.

The observation of greatest transformation induced by the 4-OH-catechol estrogen metabolites is compatible with a causal role for CYP450 1B1 in mediating cellular transformation, therefore, mRNA and protein levels for this enzyme were measured in response to treatment with E_2_. CYP450 1B1 was upregulated in response to E_2_ as demonstrated by mRNA (Figure 3A) and protein levels over the one week course of E_2_ treatment (Figure 3B). CYP450 1A1 and 1B1 catalyze estrogen oxidative hydroxylation at the C2 and C4 positions, respectively, therefore, transformation of MCF-10A cells was studied after extended treatment with 2-OHE_1_ or 4-OHE_1_, and for comparison, the parent estrogen, in this case E_1_ (Figure 1B). Treatment with 2-OHE_1_ did not significantly induce transformation, moreover, co-treatment of cells with E_1_ and the CYP450 inhibitor, α-naphthoflavone (αNF), a non-competitive CYP450 1B1 inhibitor (K*_i_* = 2.8±0.5 nM), completely ablated cellular transformation. Although α-NF inhibits other CYP450 isoforms, the simplest conclusion is that inhibition of CYP450 1B1-mediated oxidation of estrogens to the 4-OH catechols and their *o*-quinones by α-NF blocks transformation by preventing formation of these oxidative metabolites.

### Inhibition of MCF-10A cell transformation: Effects of benzothiophene SERMs and HDAC inhibitors

**Figure 4:**
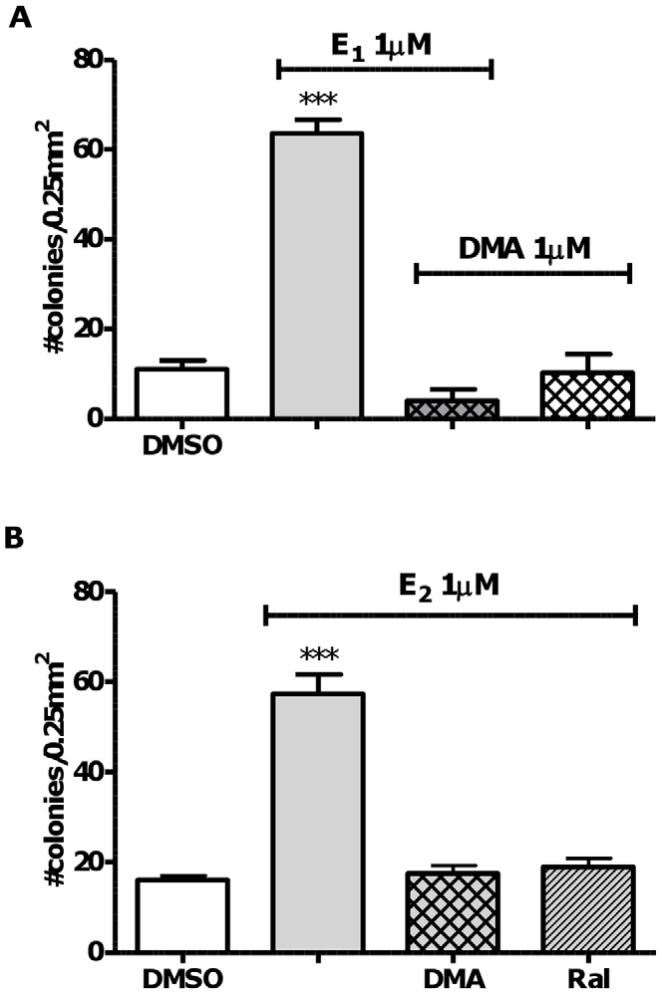
Malignant transformation induced by endogenous estrogens is inhibited by benzothiophene SERMs, but not by HDAC inhibitors. MCF-10A cellular transformation induced by E_1_ or E_2_ in the presence of benzothiophene SERMs (1 µM) was measured as anchorage-independent growth in soft agar after four weeks. **A**. DMA or **B**. DMA and the structurally related SERM, raloxifene. Cells were treated for four weeks before transfer to soft agar. Using one-way ANOVA with Dunnett's post test: *** p<0.001 versus DMSO control.

Raloxifene is clinically indicated for chemoprevention of invasive breast cancer and arzoxifene delivered similar efficacy in clinical trials [18]. Evidence for ER-independent mechanisms that may contribute to chemoprevention by benzothiophene SERMs has been reported by ourselves and others [19], [20]. HDAC inhibition has been reported to synergize actions of SERMs in breast cancer cells and HDACIs are in clinical trials [12], [21], [22]. Co-treatment of MCF-10A cells with the HDACIs, SAHA or TSA, did not attenuate estrogen-induced cellular transformation (Figure S1), therefore combinations were not explored. However, both benzothiophene SERMs, DMA and raloxifene significantly attenuated estrogen-induced cellular transformation, ablating the effects of estrogen (Figure 4A, 4B).

### Purine NCE adduct formation

**Figure 5:**
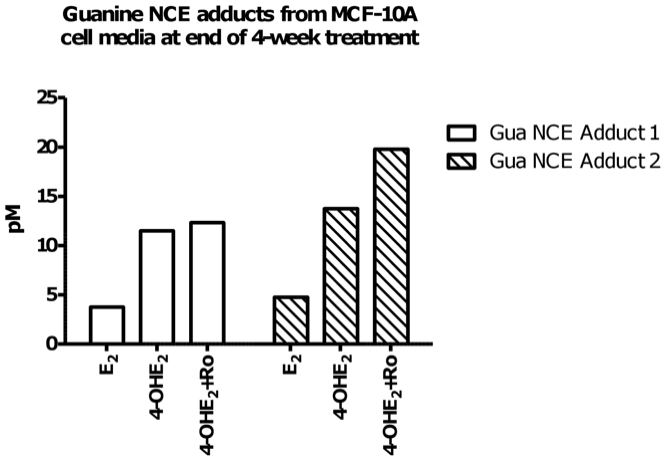
Biomarker study of guanine NCE adducts measured in supernatant of cells treated with E_2_ and catechol metabolites; guanine adducts correlate with transformation potency. Guanine NCE adducts of 4-OHE_1_ were measured in media from MCF-10A cells treated with: E_2_ 1 µM, 4-OHE_2_ 1 µM, or (4-OHE_2_ 1 µM+Ro 3 µM) at week 4. These NCEs adducts were not detectable in vehicle control experiments. N7-Me-guanine (5 nM) was used as internal standard. MRM fragmentation patterns of NCEs (m/z→m/z) corresponded to: *Gua NCE Adduct 1* 4-OHE_1_-Gua (436→152); *Gua NCE Adduct 2* 4-OHE_2_-Gua adduct (438→152).

A preliminary experiment was performed to detect purine NCE adducts in the media from MCF-10A cells at the end of the fourth week of treatment with E_2_ or 4-OHE_2_, the latter with or without COMT inhibitor (Figure 5). Media was subjected to solid phase extraction before assay by LC-MS/MS, in which MRM (multiple reaction monitoring) analysis of the appropriate mass transition (parent ion m/z and fragmentation to the appropriate purine m/z) was used to indicate the presence of the appropriate NCE adduct. N3-Me-guanine and N7-Me-guanine were used as internal standards for relative quantitation. This preliminary analysis demonstrated the presence of 4-OHE_1_-Gua, and 4-OHE_2_-Gua adducts, and adducts were not detected in vehicle treated media.

To further explore the apparent correlation between NCE adduct formation and cellular transformation, 4-OHE-1-N7Gua was synthesized and characterized spectroscopically. The ^15^N isotopologue of guanine was employed to synthesize the isotope-labeled NCE standard. Tandem MS parameters were optimized for positive MRM mode detection of Gua-NCE adducts (mass transitions m/z 438→152 and 436→152). The solid phase extraction of culture media was calculated to proceed with 70% efficiency using spiked media. Addition of ^15^N-labeled standards to media before extraction ensured accurate quantitation of NCE adducts. NCE adducts can themselves be oxidized to *o*-quinone and 8-oxo-dG adducts, therefore stability was studied over 27 h at 3<pH<10 using synthetic standards, optimal stability being observed at pH∼6.5 (Figure S2). At physiological pH in phosphate buffer, adenine NCE adduct degradation was about 80% complete after 27 h, while guanine NCE adducts in comparison were more stable. The addition of high ascorbic acid concentrations (2 mM) has been suggested to protect metabolites from oxidative degradation [23]. Stabilization of NCE adducts by citric acid and EGTA as metal ion chelators was also explored in cell culture media using the synthetic adduct standards and LC-MS/MS analysis. The highest recovery of adducts was obtained using citric acid and ascorbic acid (both 2 mM) added to the media before extraction (Figure S3).

### Attenuation of NCE adduct formation by DMA

**Figure 6:**
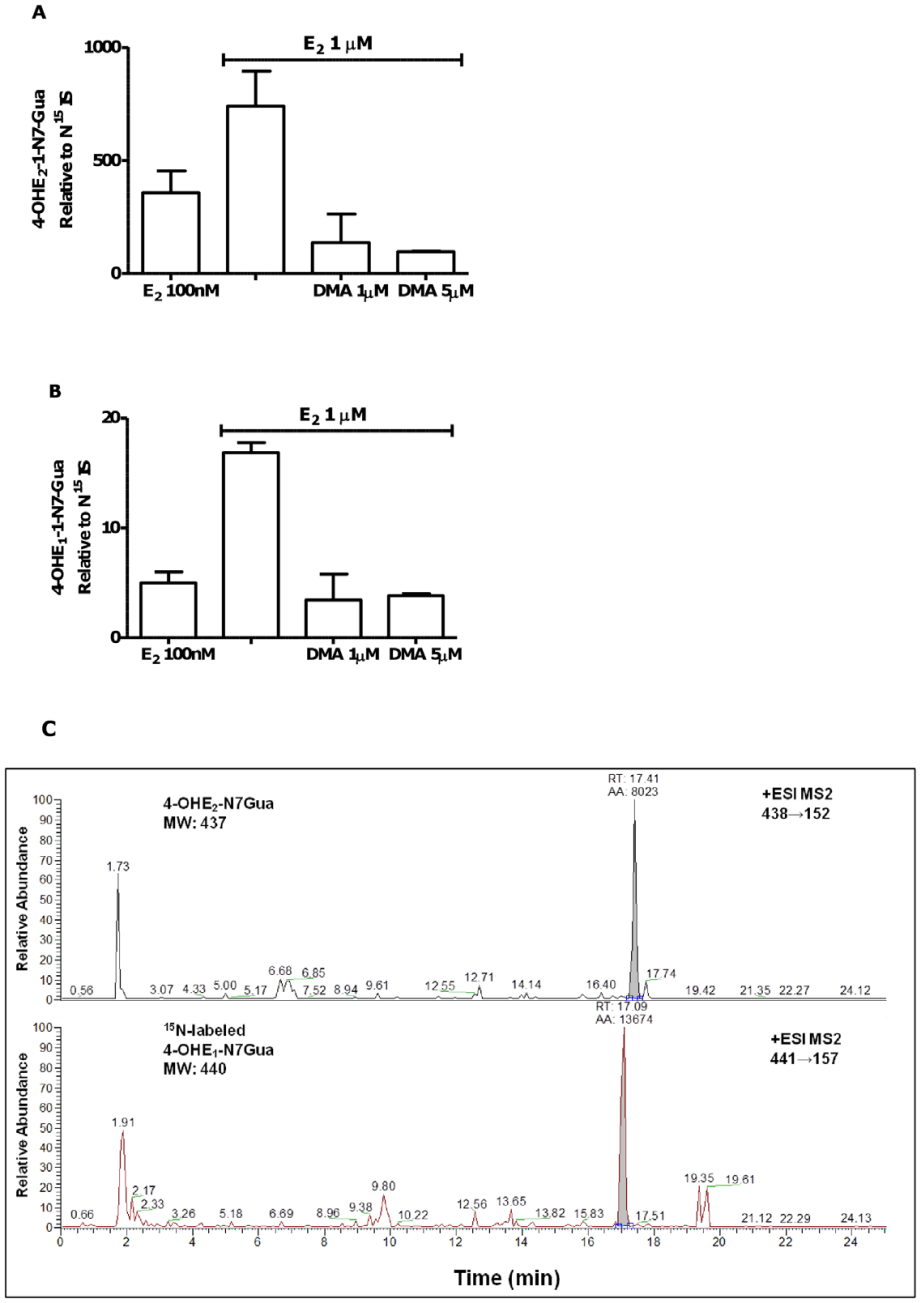
Formation of guanine NCE adducts from MCF-10A cells treated with E_2_ (0.1 or 1 µM) is inhibited by co-treatment with DMA. Cell culture supernatant was analyzed after 4 days of treatment and 1 week post cell-plating. LC-MS/MS peak area for **A**. 4-OHE_2_-1-N7Gua and **B**. 4-OHE_1_-1-N7Gua was normalized to equal amounts of internal standard ^15^N-labeled Gua adduct. **C**. Representative chromatograms of guanine NCE adducts obtained with the TSQ MS instrument. Guanine adduct 4-OHE_2_-1-N7Gua from MCF-10A cell media treated with 1 µM E_2_ after 3 days. ^15^N-labeled guanine adduct shown in red is the internal standard.

Regular administration of E_2_ with fresh media twice weekly in MCF-10A cells, and analysis of collected media (after 3 or 4 days of treatment) showed significantly increased levels of NCE adducts. Both 4-OHE_2_ and 4-OHE_1_ guanine adducts were detected (Figure 6A, 6B) and a representative measurement of 4-OHE_2_ guanine adducts is shown in Figure 6C. Levels of NCE adducts measured in media from cells treated with E_2_ were significantly reduced on co-administration of DMA (Figure 6A, 6B).

### NCE adduct formation from the nucleotide pool

**Figure 7:**
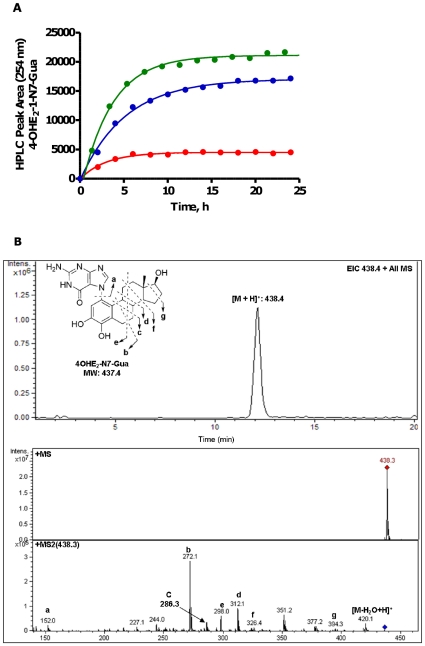
Formation of 4-OHE_2_-1-N7Gua from reaction of E_2_-3,4-Q with free Gua nucleotides. **A**. Reaction of quinone (0.2 mM) with dGMP (red), dGDP (blue), or dGTP (green) (1.9 mM) buffered at pH 6.8 (phosphate, 10 mM). Data are fit to a *pseudo*-first order exponential. **B**. Representative chromatogram of 4-OHE_2_-1-N7Gua.

Cellular concentrations of nucleotides have been measured at millimolar levels [24]. Guanosine and adenosine nucleotides play essential roles in cell function and the deoxynucleotide pool (dNTP) also provides potential substrates for NCE adduction, however, adduction of the free cellular nucleotide pools has not been previously considered as a source of the NCE adducts reported from cell culture media and human samples. The *o*-quinone of 4-OHE_2_ was freshly prepared by oxidation with MnO_2_ as previously described [25], followed by incubation with Gua and Ade nucleotides at pH 3.7 or pH 6.8. At both pHs, formation of the NCE-Gua adduct was observed from the reaction of quinone with nucleotide and subsequent depurination over the course of 24 h (Figure 7A, 7B). At pH 6.8, the amount of 4-OHE_2_-1-N7Gua generated from different nucleotides at 24 h, followed the order: dGTP>dGDP>dGMP>GTP>GDP. Since reaction of quinone with guanine is expected to be rapid, the pseudo-first order kinetics of NCE-Gua formation observed, with half-lives of ∼2–3 h, likely reflects the slower rate of depurination (Figure 7A). The depurination of NCE-adenosine nucleotide adducts was not observed from ADP and dAMP under the same conditions. The reaction of catechol estrogen quinone with the nucleotide pool and depurination to give NCE-Gua adducts is feasible under physiological conditions, at least for Gua-nucleotides and represent a source of such adducts in cell cultures and human samples.

### DNA oxidation in response to catechol estrogen treatment

**Figure 8:**
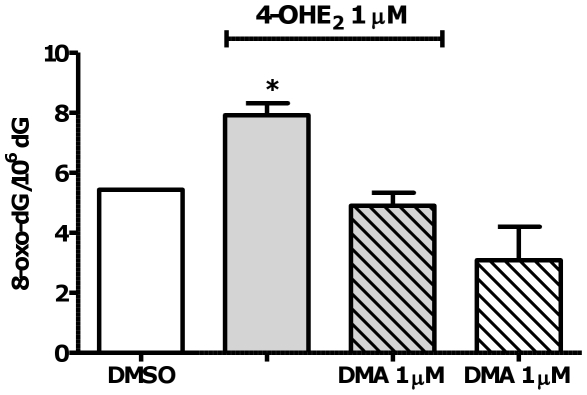
4-OHE_2_ induced oxidative damage in MCF-10A cells and DMA prevents the formation of 8-oxo-dG. Cells were treated with 1 µM 4-OHE_2_ with or without DMA 1 µM for 72 h. 8-Oxo-dG was detected and quantified by LC-MS/MS and dG was measured by HPLC. Data show mean and s.e.m. p<0.05 versus vehicle control and DMA co-treated group by ANOVA with Tukey's post test.

Surh et al. reported a fourfold increase in 8-oxo-dG levels after treatment of MCF-10A cells with 4-OHE_2_ for 6 h [26]. Herein, 8-oxo-dG was measured in DNA isolated from MCF-10A cells at a similar time point to NCE adduct measurements (3 days). In order to analytically detect 8-oxo-dG, MCF-10A cell were treated with 4-OHE_2_ (1 µM) alone or with co-administration of DMA (1 µM) for 72 h: 4-OHE_2_ induced formation of 8-oxo-dG, which was ablated by co-treatment with DMA (Figure 8).

## Discussion

Lifetime exposure to estrogens is a recognized contributor to breast cancer development [27]. Oxidative metabolism of estrogens by hydroxylation to form catechol estrogens and further oxidation to electrophilic and redox-active quinones is equally recognized to provide a potential chemical carcinogenesis contribution to mutagenesis, cancer initiation, and promotion (Figure 1) [28]. While enzyme-dependent hydroxylation is the necessary first step, cellular electrophilic and oxidative stress is dependent on rates of quinone formation and reaction, nuclear availability, cellular redox state, and detoxification by phase II enzymes. In the hamster kidney tumor model, the catechol estrogens, 4-OHE_1_ and 4-OHE_2_ were observed to be carcinogenic, whereas the corresponding 2-OH-isomers were not [29], [30]. In human and animal tissues susceptible to estrogen-induced tumors, the elevated expression of estrogen-4-hydroxylase activity is seen as support for a role for the 4-OH-catechol pathway in tumorigenesis [31], [32]. It has been reported previously that MCF-10A and MCF-10F cells undergo estrogen-induced malignant transformation, as shown by formation of anchorage-independent colonies in soft-agar media, considered a characteristic of malignant transformation [33], [34]. Since MCF-10A and 10F cells do not respond to the proliferative, hormonal actions of estrogens, these non-tumorigenic, human, mammary, epithelial cells are often classified as ER-negative and represent a model for studying estrogen-induced chemical carcinogenesis in the absence of dominant hormonal contributions. In accord with previous work, transformation of MCF-10A cells was induced by treatment with E_1_, E_2_, 4-OHE_2_, and 4-OHE_1_; whereas, the non-carcinogenic 2-OHE_1_ did not induce malignant transformation.

The catechol equine estrogen, 4-OHEN, is the major oxidative metabolite of the equine estrogens, equilenin and equilin, that constitute 50% of the widely prescribed ERT formulation, Premarin [35]. The equine estrogens, in contrast to B-ring saturated estrogens, are predominantly oxidatively hydroxylated at the 4-position, and furthermore 4-OHEN readily autoxidizes to an *o*-quinone that reacts with DNA leading to a variety of DNA lesions [36], [37], [38]. In human breast cancer cells, 4-OHEN was observed to induce DNA damage and apoptosis [39], and was toxic towards MCF-10A cells in the presence of COMT inhibitor. At lower concentrations, 4-OHEN induced malignant transformation, although colony growth was below that observed for 4-OHE_2_; induction by EN itself did not reach significance. It is tempting to link the observation of reduced transformation by equine estrogens with epidemiological data suggesting that ERT formulations based on equine estrogens are in fact safer, however, further studies are needed [40].

Two separate hypotheses have been proposed for estrogen-induced transformation of MCF-10A and MCF-10F cells. Anchorage-independent growth of MCF-10A cells induced by high concentrations of E_2_ (20 µM) was linked to CYP450 1B1 mediated oxidative metabolism, generation of ROS as a consequence of 4-OHE_2_ oxidation, and activation of NFκB [33]. Although elevation of ROS in the nucleus leading to DNA oxidation is a known potential, mutagenic pathway [33], [41], NFκB activation was argued to be causal [42]. In the present work, CYP450 1B1 was observed to be upregulated in MCF-10A cells on treatment with E_2_ and co-treatment with a CYP450 inhibitor completely attenuated malignant transformation in response to E_1_. This observation confirms a role for oxidative metabolism and suggests that transformation results from induction of cellular oxidative or electrophilic stress by the 4-OH catechol estrogen. Elevated levels of 8-oxo-dG were also observed, confirming induction of oxidative stress leading to DNA oxidation.

The second hypothesis posits DNA damage via formation of NCE adducts as causal in malignant transformation, the foundation being comparison of levels of NCE adducts to stable DNA adducts after reaction of estrogen quinones with DNA [43]. Stable adducts detected by P^32^-postlabeling analysis were reported to be 10–50 fold more abundant from reaction with 2-OH estrogen catechol quinones, incompatible with the lower carcinogenicity of 2-OHE_2_ versus 4-OHE_2_ [17], [44], [45]. The predominant formation of NCE adducts from 4-OH estrogens was interpreted as evidence for a causal role for 4-OHE-NCE adducts in carcinogenesis [46]. Furthermore, 4-OHE-1-N7Gua NCE adducts were detected from female Sprague-Dawley rats treated with 4-OHE_2_ at levels orders of magnitude greater than stable adducts detected by P^32^-post-labeling [47], underpinning the theory of formation of abasic sites by depurination of DNA as the cause of estrogen-induced mutagenesis [23], [48], [49], [50]. However, abasic sites are a natural intermediate in base excision repair and are present at very high levels in normal cells [51]. In the present work, 4-OHE-1-N7Gua was measurable by LC-MS/MS analysis of supernatant from incubations of MCF-10A cells with either E_2_ or 4-OHE_2_ with and without COMT inhibitor. However, production of 4-OHE_2_-1-N7Gua was observed from the reaction of the estrogen 3,4-*o*-quinone with dGTP, dGDP, dGMP, GTP, and GDP, with the highest yield from dGTP. The generation of *o*-quinones from CYP450-mediated metabolism of estrogens, in the presence of cellular nucleotide pools that may be at millimolar levels, raises the possibility that these may be the source of NCE adducts. Indeed, adduction of dNTP and incorporation into DNA has been considered as a mutagenic mechanism [52]. Therefore, the alternative hypothesis must be considered that 4-OHE_2_-1-N7Gua represents a quantitative biomarker of cellular electrophilic stress from the free nucleotide pool.

Estrogen-induced malignant transformation of MCF-10A cells, measured by anchorage-independent growth, was inhibited by the SERMs raloxifene and DMA. In addition to potential clinical relevance, this observation allowed testing of the correlation of biomarkers of oxidative and electrophilic stress with attenuated transformation. Prevention by DMA was shown to be accompanied by significant attenuation of NCE adduct formation and also by blockade of estrogen-induced 8-oxo-dG formation.

The detailed mechanism by which raloxifene and DMA attenuate malignant transformation requires further study. Classical ER-dependent mechanisms are unlikely to be effective, since the antiestrogen ICI 182780 was previously shown to be ineffective in inhibition of the transformation phenotypes induced by E_2_ and 4-OHE_2_ [44]. The phenolic antioxidant resveratrol has been reported in MCF-10 cells to inhibit estrogen-induced malignant transformation [34], [53], [54]. Furthermore, a quinone-forming catechol metabolite of resveratrol, was reported to inhibit estrogen-induced malignant transformation in MCF-10A cells by reaction of the quinone with essential cysteine residues in IKK kinases [64]. Both raloxifene and DMA are phenolic antioxidants; are oxidatively activated to quinones that have been shown to modify protein-cysteine residues; and, have demonstrated ER-independent chemopreventive activity [19], [55], [56], [57].

### Conclusions

This is the first report that the benzothiophene SERMs, raloxifene and DMA, block estrogen-induced malignant transformation of human breast epithelial cells. This observation and that of lower transformation by equine estrogen components of ERT may of clinical relevance. Attenuated cellular transformation by DMA allowed correlation with biomarkers of cellular oxidative and electrophilic stress, 8-oxo-dG and NCE adducts, respectively. CYP450 oxidative metabolism of estrogens is required to induce malignant transformation that is accompanied by oxidative and electrophilic stress, and the formation of NCE adducts may occur from reaction of quinones with free nucleotide pools, which has not previously been considered.

## Materials and Methods

### Materials and Reagents

E_1_/E_2_, EN, 4-OHE_1_/E_2_, 2-OHE_1_ estrogens were obtained from Steraloids Inc. (Newport, RI). αNF, Ro 41-0960 (Ro), solvents, reagents and other synthesis chemicals were obtained from Sigma (St. Louis, MO). SAHA and TSA were obtained from Cayman Chemical (Ann Arbor, MI). DMA was synthesized as described previously [58]. 4-OHEN was synthesized by treating equilin (EQ) with Fremy's salt as described previously [32] with minor modifications [37].

### Cell Culture

All cell culture reagents were purchased from Invitrogen (Carlsbad, CA) unless stated otherwise. MCF-10A cells were a kind gift from Dr. Meiling Chen (UIC), originally from MCF. MCF-10A cells are grown in phenol red free (PRF) D-MEM/F-12 media supplemented with 100 ng/mL cholera toxin, 10 g/mL insulin, 0.5 g/mL hydrocortisol, 20 ng/mL EGF, 1% 10,000 U penicillin G, 10 mg/mL streptomycin, and 5% stripped FBS. Estrogen-free media was prepared by supplementing 3× dextran-coated charcoal-treated FBS. MCF-10A cells were treated with each compound (1 µmol) twice weekly for four weeks. Cells were passaged weekly or as needed throughout the four-week treatment. For DNA oxidation experiments, MCF-10A cells were plated in 15 cm-dishes at 2×10^6^ cells density. Cells were allowed to attach overnight and then were treated with 4-OHE_2_ with DMA for 72 h.

### Sample Preparation

For solid phase extraction of NCE adducts, media was collected every 3 or 4 days at which time ascorbic acid (AA) 2 mM and citric acid (CA) 2 mM were added to minimize oxidative degradation of NCE adducts in the media based on preliminary stability studies. Media was analyzed immediately or stored at −20°C. In order to analyze the media for formation of depurinating adducts, ice-cold acetone was added to induce protein precipitation and possible dissociation of the lipophilic depurinating adducts from media proteins. Before further manipulation, known amounts of stable isotopically ^15^N-labeled internal standards of adenine and guanine adducts were added into the media. The media and acetone mixture was stored at −20°C for 30 min and centrifuged at 10 K for 15 min. Acetone was removed from supernatant by nitrogen bubbling. NCE adducts were extracted by a C8 gravity column (Bond Elut, Varian), followed by elution with MeOH/H_2_O/TFA (8∶1∶0.1), or ACN/MeOH/H_2_O/FA (8∶1∶1∶0.1). After drying, samples were dissolved in MeOH.

DNA extraction for 8-oxo-dG analysis was run as previously described [59] with minor modifications. The cells were washed with ice-cold PBS (10 mM, pH 7.4) and collected by scraping in 4 mL PBS final volume. The collected cells were centrifuged at 1,500 rpm for 5 min. After centrifugation, the cell pellets were homogenized in 1 mL of lysis buffer (320 mM sucrose, 10 mM Tris, pH 7.4, 5 mM MgCl_2_, 10 mM Triton X-100, and 50 mM mannitol). The nuclei pellets were treated for 30 min at 37°C with RNase T1 (1000 units) and RNase A (0.2 mg, 2 units) in 997 µL solution buffer (1% SDS, 1 mM EDTA, 10 mM Tris, pH 7.4, 0.45 M NaCl) and further incubated with proteinase K (0.8 mg) for 30 min at 37°C. After cooling down the samples at 4°C, NaCl (5 M, 171 µL) and Tris (1 M, 14.2 µL, pH 7.4) were added to achieve final concentration of 0.62 M and 20 mM, respectively. After 1 mL of chloroform/isoamyl alcohol mixture (24∶1, v/v) was added, the samples were thoroughly mixed by vortex for 20 sec and centrifuged at 4,000 rpm for 15 min, followed by isolation of the upper aqueous layer. Then an equal volume 1∶1 (v/v) of ice-cold isopropanol was added, gently mixed by inverting and kept at −20°C for 30 min for DNA precipitation. The DNA was washed three times with 70% ethanol. Finally, DNA was isolated in 100 µL of 25 mM ammonium acetate buffer (pH 6.8) containing 1 mM ZnCl_2_ and 10 mM MgCl_2_. The DNA concentrations were calculated by measuring the absorbance at 260 nm according to the ratio of one absorbance units equals 50 µg/mL of DNA. DNA hydrolysis was carried out in DNA solution mixed with 1 µL of 10 mM deferoxamine and 0.5 µL of 10 mM BHT, and hydrolyzed by incubation with DNase 1 (20 units, in a reaction buffer containing Tris, pH 7.4, 1 mM MgCl_2_) and nuclease P_1_ (10 units) at 55°C for 3 h. The mixture was further incubated with alkaline phosphatase from calf intestine (15 units), phosphodiesterase I (1 unit, in a reaction buffer containing 10 mM Tris, pH 8) and 5 µL of MgCl_2_ (10 mM) at 37°C for 3 h. After incubation, 10 µL of ammonium acetate (0.1 M, pH 5.3) was added to neutralize solution. Stable isotopically labeled ^15^N_5_8-oxo-dG was added to the solution as internal standard. The mixtures were then centrifuged for 15 min at 13,000 r.c.f. using Costar 0.22 µm nylon filters.

### MS Instrumentation and HPLC

The NCE LC/MS/MS analysis was completed by either of two methods. (1) An Agilent API-3000 (Applied Biosystems) triple-stage quadrupole mass spectrometer utilizing the electrospray ionization method coupled to a Shimadzu LC-1-AD HPLC system and utilizing the electrospray ionization method. Adducts were resolved by HPLC on a Waters X-Bridge C18 column (3.5 µm; 2.1 mm×150 mm) at a flow rate of 0.2 mL/min with a 5 min linear gradient of A (acetonitrile, ACN and 0.1% formic acid, FA) and B (H_2_O and 0.1% FA) from 10% to 60%, then 60% to 90% in 8 min, and then holding constant 90% A for an additional 3 min. The column was returned to original conditions in 2 min, and allowed to equilibrate for another 8 min under initial conditions. (2) A TSQ quantum triple quadrupole mass spectrometer (Thermo Finnigan) instrument coupled to HPLC, while adducts were resolved with an X-Bridge phenyl column (3.5 µm; 2.1 mm×150 mm) at a flow rate of 0.2 mL/min with a 4 min linear gradient of A (ACN) and B (H_2_O and 0.1% FA) from 20% to 60%, then 60% to 85% in 7 min, then 85% to 90% in 1 min, and then holding constant 90% A for an additional 3 min. The column was returned to original conditions in 3 min. Adducts were confirmed against N^15^-labelled guanine and adenine adducts. The collision energies were optimized at 55 volts for guanine adducts and 53 volts for adenine adducts. Samples were detected using ESI positive MRM mode carried out at 350°C. In the TSQ instrument, collision energies were optimized to 47 and 50 volts for E_1_ and E_2_ adenine adducts respectively, and 49 and 50 volts for E_1_ and E_2_ guanine adducts respectively.

The 8-oxo-dG LC/MS/MS method was completed on API 3000 (Applied Biosystem, Foster City, CA, USA) triple quardrupole mass spectrometer attached to Agilent 1200 HPLC (Agilent Technologies, Santa Clara, CA). First, stable isotopically labeled ^15^N_5_8-oxo-dG was added to the solution as internal standard. The samples were separated using a Phenomenex Kinetex C18 column (3×100 mm, 2.6 µm) and ADV-FFKIT filter (Analytical, Prompton Plains, NJ, USA) at a flow rate of 0.3 mL/min with a gradient mobile phase starting 10% methanol for 1 min, and increasing to 40% methanol over 4 min, increasing to 50% methanol over 3 min, increasing to 60% methanol over 5.5 min, keeping to 60% for 1.5 min and then equilibrium with 10% methanol for 9 min. The native dG was determined by HPLC (UV) scanning from 280 nm. The 8-oxo-dG was detected multiple reaction monitoring and collision-induced dissociation for the fragmentation pathway of *m/z* 284→168 and *m/z* 289→173 for ^15^N_5_8-oxo-dG using positive ion electrospray.

### Synthesis of Standards

For NCE standards, E-3,4-Q was prepared by MnO_2_ catalyzed oxidation in CHCl_3_ as described previously with minor modifications [25]. To a solution containing 4-OHE_1_ (8 mg, 0.028 mM) in dry CHCl_3_ (1.5 mL) at −30°C was added activated MnO_2_ (25 mg). The reaction was stirred for 10 min and the solution was filtered. The resulting solution was evaporated in nitrogen atmosphere at −30°C. The resulting brown solid was dissolved in an equal volume of DMF or acetonitrile. Reaction of E-3,4-Q with deoxyguanosine (dG) and deoxyadenosine (dA): the synthesis was carried out as reported previously [60]. Briefly, to a solution containing 2′-deoxyguanosine (30 mg, 0.112 mM) or 2′-deoxyadenosine (30 mg, 0.119 mM) in 1 mL of acetic acid/water (50/50, v/v) was added E-3,4-Q (8 mg, 0.028 mM) and the mixture was stirred at room temperature for 4 hours. The reaction mixture was filtered and the nucleic acid adduct of E_1_ was purified by reverse phase HPLC (20 mm×250 mm, 100 Å C_18_ column; flow rate 5.0 mL/min, mobile phase 5% to 90% acetonitrile gradient in water over 25 min, held at 90% for 15 min.) Guanine adduct 4-OHE_1_-1-N7Gua (3 mg, 0.007 mM) was obtained as a solid and E_1_-adenine adduct was not formed in this reaction. Above procedure was followed to synthesize ^15^N_5_-guanine adduct. Reaction of E-3,4-Q with adenine: the synthesis was carried out as reported previously [61], [62], [63]. To a reaction mixture in DMF containing sodium dithionite (15 mg, 0.086 mM) and adenine nucleic acid base (0.22 mM) was added E-3,4-Q (8 mg, 0.028 mM) and the mixture was stirred at room temperature under nitrogen for 45 min. The reaction mixture was filtered and evaporated DMF *in vacuo*. The adenine nuclear base adduct-E_1_ was purified by reverse phase HPLC (20 mm×250 mm, 100 Å C_18_ column; flow rate 5.0 mL/min, mobile phase 5% to 90% acetonitrile gradient in water over 25 min, held at 90% for 15 min.) Adenine adduct 4-OHE_1_-1-N3Ade was isolated. Above procedure was also followed to synthesized ^15^N_2_-adenine adduct. For 8-oxo-dG standard, stable isotopically labeled ^15^N_5_-8-oxo-dG was synthesized as described previously [64].

### Kinetic study of NCE adduct formation from free nucleotide pool

All stock solutions of nucleotides (dGMP, dGDP, dGTP, GTP, GDP, dAMP, ADP) were prepared freshly before the initiation of the reaction in either pH 6.8 10 mM phosphate buffer or pH 3.7 10 mM citric buffer. The stock solution of E_2_-3,4-Q (ca. 4 mM) was made in a mixture of ACN/DMF (3/1 v/v) and stored at −20°C. The reaction was initiated by mixing 25 µL stock solution of E_2_-3,4-Q with 475 µL of 2 mM nucleotide solution in buffer that was pre-incubated at 37°C for 10 min. The initial concentrations of E_2_-3,4-Q and nucleotide in the reaction mixture were obtained as 0.2 mM and 1.9 mM respectively. Reaction mixture was kept at 37°C and monitored by HPLC (Shimadzu) with UV detection at 254 nm and 280 nm for 24 hrs. The formation of 4-OHE_2_-1-N7Gua was confirmed by comparing with the synthetic standard using HPLC and LC-MS/MS (Agilent, Ion-trap). The kinetics of the formation of E_2_-guanine adduct was studied by measuring the changes in the HPLC peak area of 4-OHE_2_-1-N7Gua as a function of time. The LC condition used for both HPLC and LC-MS/MS is as follows: Analytical Advantage ARMOR C18 column, (5 µm, 4.6 mm×150 mm), flow rate 1.0 mL/min, the elution program started with 30% of B (MeOH) for 5 min then gradient to 98% in A (H_2_O with 10% of MeOH) over 20 min, held at 98% of B for 8 min, returned to 30% of B in 5 min and held for an additional 2 min.

### Anchorage Independent Growth in Soft Agar

After the 4-week treatment, cells were seeded at 50,000 cells/well density in triplicates in 12-well plates. Cells were suspended in 1 mL of 0.35% soft agar in media, over 1.5 mL of 0.7% soft agar. Colonies of transformed cells were allowed to grow for another four weeks at 37°C and 5% CO_2_. Anchorage-independent growth (spherical formation of ≥10 cells) was scored using a light microscope, by counting foci in 0.25 mm^2^ areas [65].

### Western Blots and PCR

MCF-10A cells were treated with E_2_, EN, and E_2_+DMA, 1 µmol each for various incubation times as indicated. Cells were trypsinized, pelleted, washed in PBS, resuspended in IP buffer (50 mM HEPES, 150 mM NaCl, 1 mM EDTA, 2.5 mM EGTA, 10 mM β-glycerophosphate, 10% glycerol, and 0.5% NP-40, *p*H = 8.0) containing protease inhibitors, mixed, and centrifuged at 12,000 *g* for 10 min. Protein concentration was measured in supernatants using the Bradford assay kit (Bio-Rad Laboratories). Equal aliquots of total protein samples (20 µg per lane) were electrophoresed on a 4–12% Bis-Tris polyacrylamide gel, transferred to PVDF membranes (Millipore, Bedford, MA), and blotted using antibodies to CYP450 P450 1B1 from Santa Cruz Biotechnology (Santa Cruz, CA). β-actin antibody was from Cell Signaling Technology (Beverly, MA); it was used as a control for loading and transfer. The blotted proteins were visualized using the enhanced chemiluminescence detection system from Amersham Biosciences (Piscataway, NJ) and quantitated using DT software.

For PCR, MCF-10A cells were plated in 100 mm dishes and treated with vehicle control DMSO, E_2_ 1 µmol for various incubation times as indicated. mRNA was collected according to standard Trizol method (manufacture's protocol, Invitrogen). For quantitative PCR (experiment run in Jonna Frasor's Lab, UIC), the primers used for CYP450 P450 1B1 were forward 5′ CATGAGTGCCGTGTGTTTCG and reverse 5′ TCTTCGTTGTTGGCTGAGCAG. One µg of total RNA was reverse transcribed using Moloney murine leukemia virus reverse transcriptase. The resulting product was diluted to 200 µL with double-distilled H_2_O, and 2 µL were used for each subsequent QPCR. QPCR was carried out and analyzed as previously described [66].

### Statistics

The data were reported as the mean ± S.E.M. One-way ANOVA analysis with Tukey's multiple comparison test was done using Graph-Pad Prism version 4.00 for Windows, GraphPad Software.

## Supporting Information

Figure S1 **Malignant transformation induced by endogenous estrogens is not inhibited by cotreatment with HDAC inhibitors.** MCF-10A cellular transformation induced by E_2_ 1 µM in the presence of HDAC inhibitors, suberoylanilide hydroxamic acid (SAHA) and trichostatin A (TSA) tested at 100 nM each. Cells were treated for four weeks before transfer to soft agar. Using one-way ANOVA with Dunnett's post test: *** p<0.001 versus DMSO control.
(TIF)

Figure S2 **Stability of depurinating adducts at various** ***p*** **Hs incubated for 4, 16 and 27 h.** Known concentrations of 4-OHE_1_-1-N3Ade in **A**. and 4-OHE_1_-1-N7Gua in **B**. were incubated in PBS buffer at different pHs. Data shown represent the MS signal normalized to the universal highest signal detected in the study for each adduct.
(TIF)

Figure S3 **Stability and proper storage of depurinating adducts.** **A**. Stability of 4-OHE_1_-1-N3Ade incubated in cellular media at 37°C for 24 h and extracted after the addition of AA 2 mM alone or together with 2 mM EGTA and CA 2 mM. **B**. Stability of 4-OHE_1_-1-N7Gua measured by the API triple quadrupole MS by quantifying the peak height (PH). **C**. Similar results were obtained by measuring the 4-OHE_1_-1-N7Gua, in the TS quantum MS by quantifying the peak area (PA). The presence of both AA and CA increased the amount depurinating adducts recovered. All data is normalized to equimolar internal standard ^15^N-labeled 4-OHE_1_-1-N7Gua.
(TIF)
