# Supplementary figures and images for: Raloxifene and Desmethylarzoxifene Block Estrogen-Induced Malignant Transformation of Human Breast Epithelial Cells
Source: PLoS One. 2011 Nov 29;6(11):e27876. doi: 10.1371/journal.pone.0027876 (PMC3226622; doi:10.1371/journal.pone.0027876)

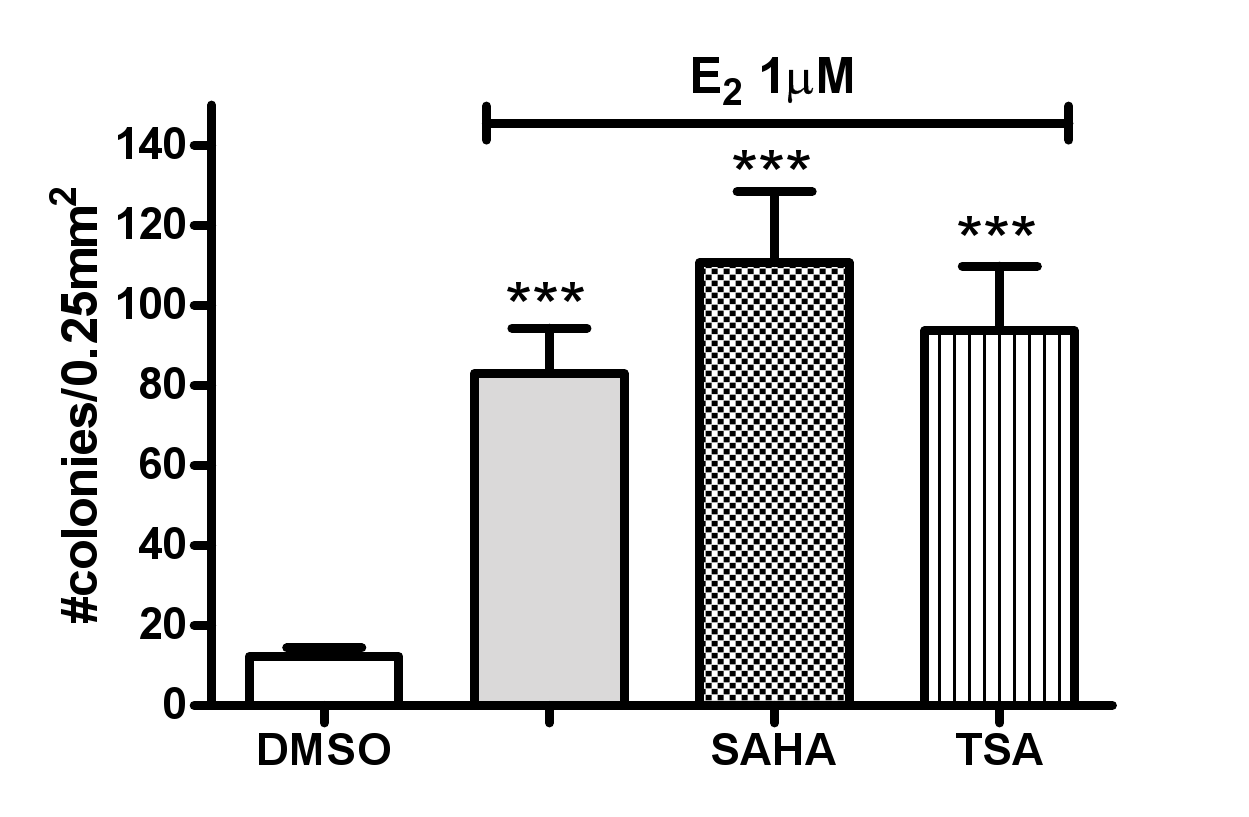

Supplement: Figure S1 — Malignant transformation induced by endogenous estrogens is not inhibited by cotreatment with HDAC inhibitors. MCF-10A cellular transformation induced by E2 1 µM in the presence of HDAC inhibitors, suberoylanilide hydroxamic acid (SAHA) and trichostatin A (TSA) tested at 100 nM each. Cells were treated for four weeks before transfer to soft agar. Using one-way ANOVA with Dunnett's post test: *** p<0.001 versus DMSO control. (TIF) [file pone.0027876.s001.tif]

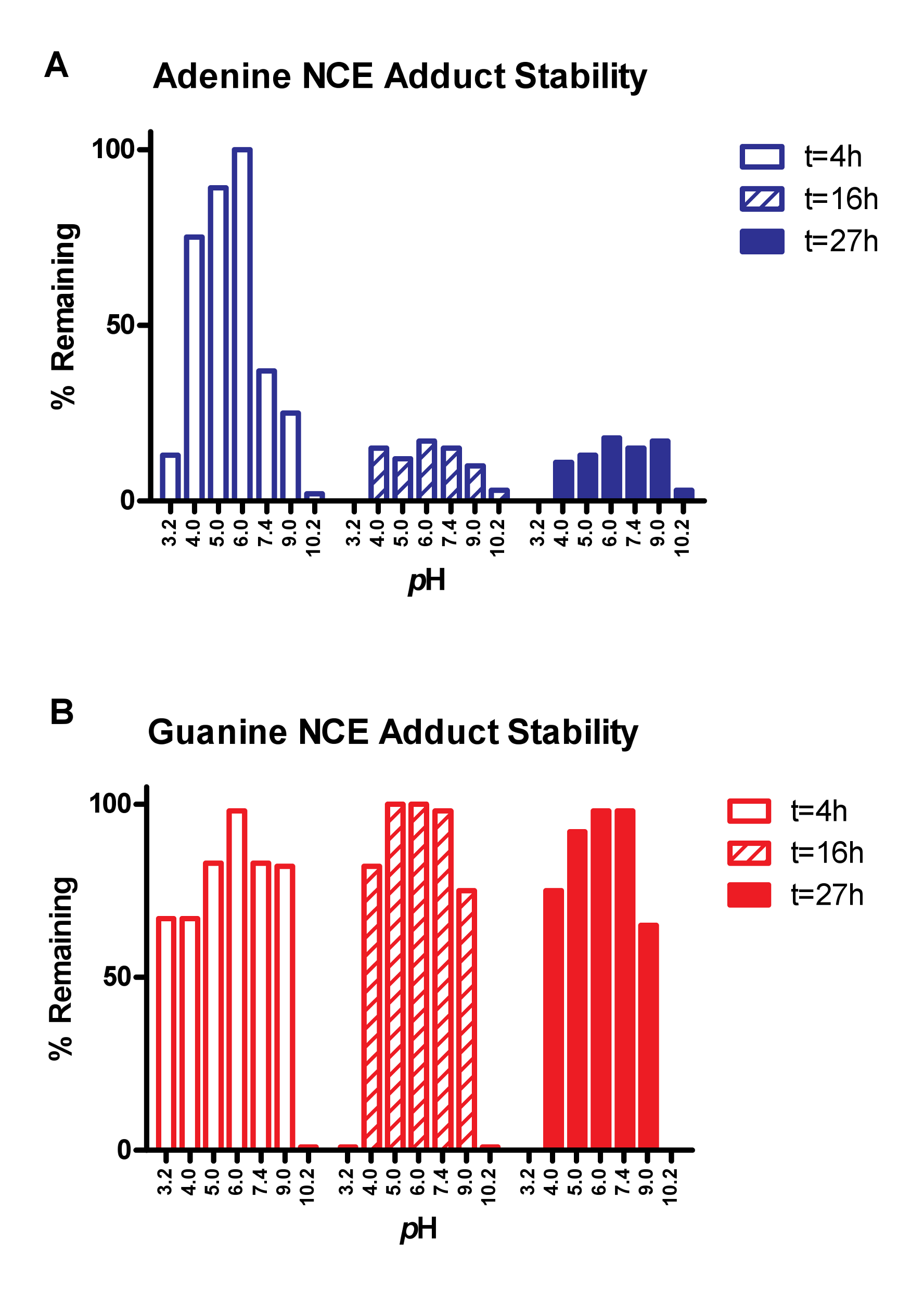

Supplement: Figure S2 — Stability of depurinating adducts at various p Hs incubated for 4, 16 and 27 h. Known concentrations of 4-OHE1-1-N3Ade in A. and 4-OHE1-1-N7Gua in B. were incubated in PBS buffer at different pHs. Data shown represent the MS signal normalized to the universal highest signal detected in the study for each adduct. (TIF) [file pone.0027876.s002.tif]

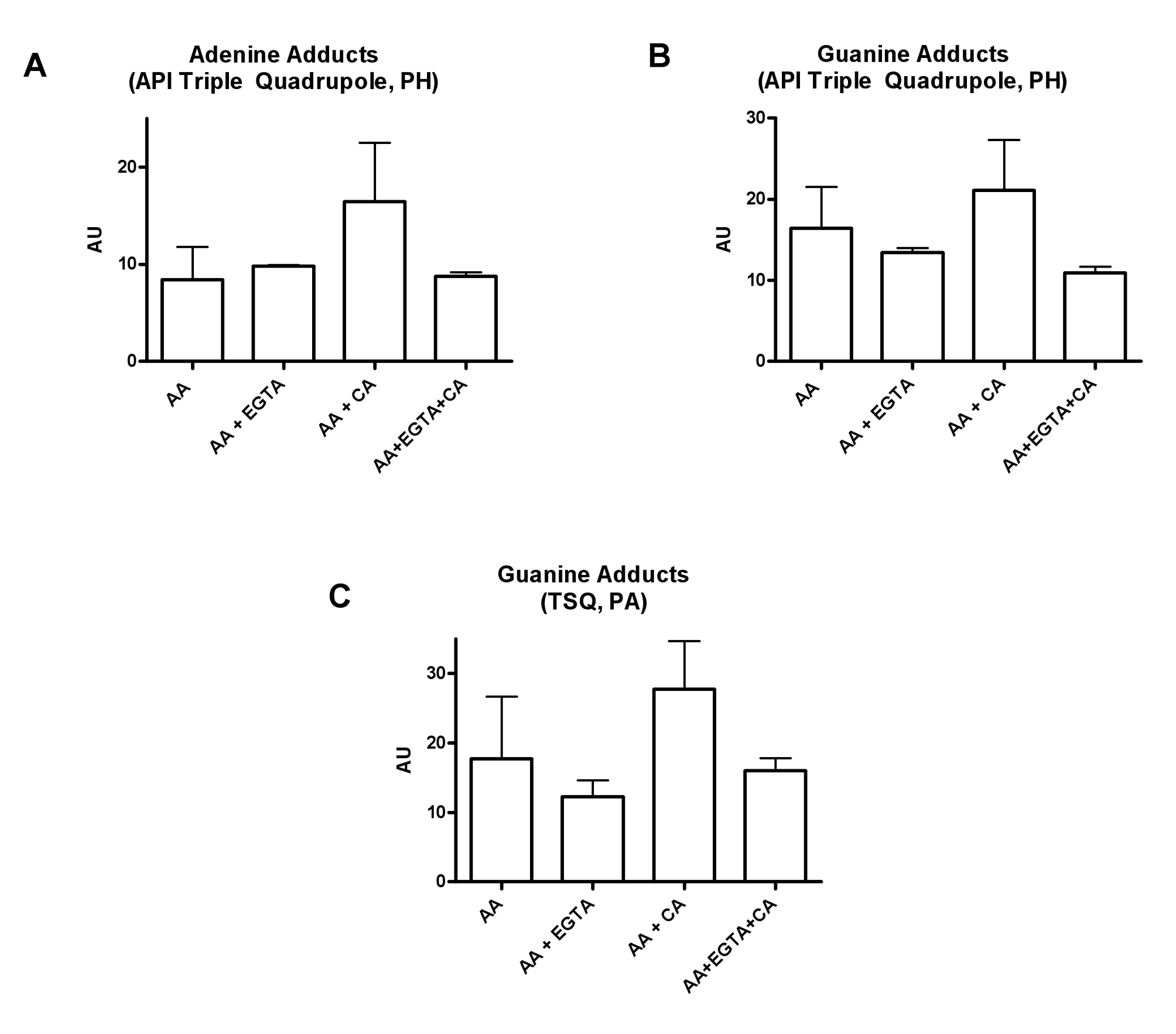

Supplement: Figure S3 — Stability and proper storage of depurinating adducts. A. Stability of 4-OHE1-1-N3Ade incubated in cellular media at 37°C for 24 h and extracted after the addition of AA 2 mM alone or together with 2 mM EGTA and CA 2 mM. B. Stability of 4-OHE1-1-N7Gua measured by the API triple quadrupole MS by quantifying the peak height (PH). C. Similar results were obtained by measuring the 4-OHE1-1-N7Gua, in the TS quantum MS by quantifying the peak area (PA). The presence of both AA and CA increased the amount depurinating adducts recovered. All data is normalized to equimolar internal standard 15N-labeled 4-OHE1-1-N7Gua. (TIF) [file pone.0027876.s003.tif]
